# Congenital pulmonary arteriovenous malformation: a rare cause of cyanosis in childhood

**Published:** 2009-11-08

**Authors:** Hassan Mottaghi, Mahdi Kahrom, Mohammad Hassan Nezafati, Hadi Kahrom

**Affiliations:** 1 Division of Cardiology, Department of Pediatrics, Imam Reza Hospital,; 2 Department of Cardiac Surgery, Imam Reza Hospital, Mashhad University of Medical Sciences, Mashhad, Iran

**Keywords:** Pulmonary arteriovenous malformation, Cyanosis, Right to left shunting

## Abstract

Pulmonary arteriovenous malformation (PAVM) is a rare condition in which there is abnormal connection between pulmonary arteries and veins. The disorder usually appears in late childhood or early adult life, with dyspnea on exertion, clubbing or cyanosis. We present two patients with severe cyanosis and their work-up to diagnosis of PAVM, as a rare cause of cyanosis in childhood.

## Background

Pulmonary arteriovenous malformations (PAVMs) are caused by abnormal communications between pulmonary arteries and pulmonary veins, which are most commonly congenital in nature. Although these lesions are quite uncommon, they are an important part of the differential diagnosis of common pulmonary problems such as hypoxemia, pulmonary nodules and cyanosis. PAVM is a rare disorder with an incidence of 2–3 per 1000000 population [1]. It occurs twice as often in women as in men, but there is a male predominance in newborns [2]. Around 10% cases of PAVM are identified in infancy or childhood, followed by a gradual increase in the incidence through the fifth and sixth decades. Approximately 70% of the cases of PAVM are associated with hereditary hemorrhagic telangiectasia (HHT). Conversely, about 15 to 35% of patients with HHT have PAVM [3]. PAVMs may cause hypoxemia and dyspnea due to right to left shunting, but remain frequently undiagnosed. This intrapulmonary malformation is described in two patients who presented with severe cyanosis.

## Patients and case reports

### Patient 1

A 6-month-old male infant presented to our hospital with cardiac murmur and progressive cyanosis of one day. He was born at term after a normal pregnancy and without prenatal complications. His family history was unremarkable and specifically negative for cardiopulmonary disorders. He had dusky color, but was relatively well and thriving. His mother related a history of orthodeoxia with an increase in cyanosis when her infant is embraced and decrease in cyanosis when lying on the back.

Cardiovascular examination revealed bounding peripheral pulses in both upper and lower limbs, normal first and second heart sounds and a continuous machinery murmur, which were heard at the left upper sternal border space, suspicious of patent ductus arteriosus (PDA). Chest examination revealed normal vesicular breathing, equal on both sides of the chest. The examination of abdomen, central nervous system, skin and mucosa did not reveal any abnormality.

Investigations showed arterial blood gases (room air) to have pH of 7.23, PCO _2_ 37mmHg, HCO _3_ 17mmol/L, PO _2_ 34 mm Hg and O _2_ saturation 61%. Chest X-ray showed normal cardiac size and clear fields in both lungs. Electrocardiogram showed normal sinus rhythm, normal QRS axis, and no right ventricular hypertrophy. Other laboratory findings showed hemoglobin levels of 11.8 g/dl with normal methemoglobin of 1.2%.

**Figure 1:**
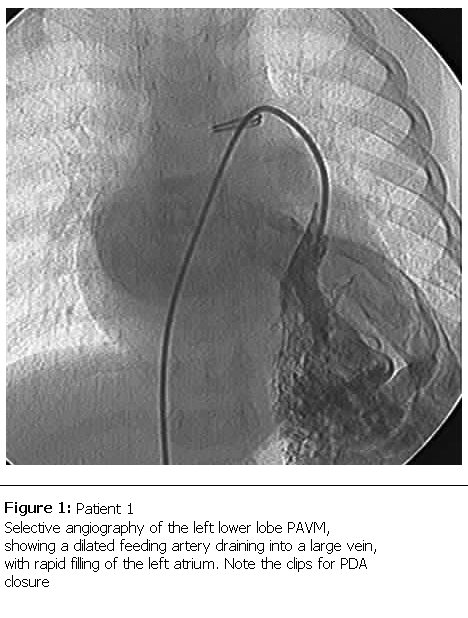
Patient 1 Selective angiography of the left lower lobe PAVM, showing a dilated feeding artery draining into a large vein, with rapid filling of the left atrium. Note the clips for PDA closure

Echocardiography confirmed the PDA in our patient, but was not a rationale for his diagnosed cyanosis. Our patient underwent video assisted thoracoscopic surgery (VATS) for closure of PDA. To our expectation, as the PDA closure did not resolve the progressive cyanosis and early clubbing, contrast echocardiography study was performed, suggesting the presence of PAVM with significant right to left shunt, as evidenced by rapid filling of the left atrium with dissolved bubbles. Cardiac catheterization performed which revealing the pulmonary arteriovenous malformation in postero-basal segment of lower zone of the left lung ( Figure 1 ), with normal pulmonary artery pressure of 15/5mmHg (mean 10). Oxygen saturation of the aorta was 64%, which made him a candidate for surgical versus catheter-based intervention. Percutaneous transcatheter embolization of PAVM with spring coils was performed in this patient with significant improvement in systemic oxygenation and excellent result in 5 years follow up.

### Patient 2

A 10-year-old girl was referred to the department of pediatric cardiology with severe cyanosis. There was a history of bluish discoloration of fingers and lips for the past 5 years. There was no history of breathlessness. On examination, the patient was thin built; peripheral pulses were normal, blood pressure 95/72 mmHg, with normal S1 and S2. There was central cyanosis in lips and nails and grade 2 clubbing was present. There was no evidence of mucocutanious telangiectasis. Examination of cardiopulmonary and nervous system was essentially normal. Hemoglobin was 16.8g/dl with normal methemoglobin of 2%. Chest X-ray and pulmonary function tests (PFTs) were reported to be normal by pulmonologists. Arterial blood gas analysis at room air showed pH 7.20, PO _2_ 58.8 mmHg, PCO _2_ 40.3 mmHg and oxygen saturation of 88%.

**Figure 2:**
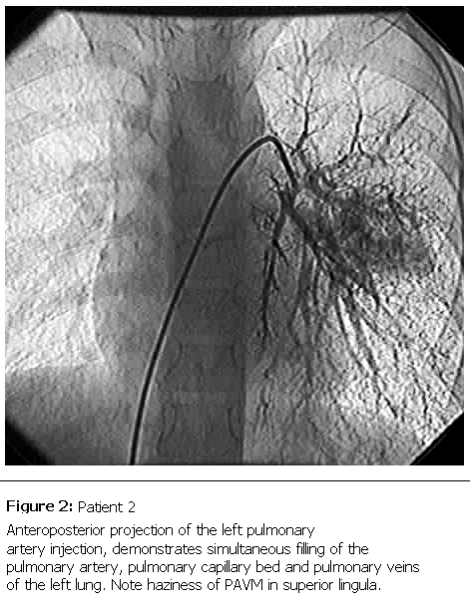
Patient 2 Anteroposterior projection of the left pulmonary artery injection, demonstrates simultaneous filling of the pulmonary artery, pulmonary capillary bed and pulmonary veins of the left lung. Note haziness of PAVM in superior lingula.

Contrast echocardiographic study was performed, but did not reveal any significant anomaly. Cardiac catheterization was performed and injection of contrast media in the main pulmonary artery showed immediate opacification of the left atrium and small haziness in the left lung. Selective injection of contrast media in left pulmonary artery demonstrated the PAVM in superior lingula of the left lung ( Figure 2 ). Successful transcatheter embolization was attempted in the patient and resulted in resolution of cyanosis during 6 moths follow up.

## Discussion

Pulmonary arteriovenous malformation (PAVM) is a rare cardiovascular anomaly. Most cases are congenital, frequently related to hereditary hemorrhagic telangiectasia (HHT), an autosomal dominant disorder. Abnormal communications between blood vessels of the lung may also be found in a variety of acquired conditions such as hepatic cirrhosis, schistosomiasis, mitral stenosis, trauma, actinomycosis, Fanconi’s syndrome and metastatic thyroid carcinoma [4, 5]. Fifty-three to seventy percent of PAVMs are found in lower lobes, 75% of patients have unilateral disease, 36% have multiple lesions, and half of those with multiple lesions have bilateral disease [6]. PAVMs are supplied by pulmonary arteries in about 95% of cases, and by systemic arteries less frequently [7].

Symptoms in early life may vary from being totally absent to severe cyanosis, congestive heart failure and even fulminant respiratory failure [2]. Symptoms related to PAVM often develop between the 4th and 6th decades. Progression of PAVMs is often noted during pregnancy [8]. A progressive increase in cyanosis is often noted because of the opening of new or enlargement of the existing fistula, and development of polycythemia. Haemoptysis and haemothorax may occur, especially in patients with HHT and in pregnant women. Neurologic sequels are quite common and may occur in up to 33% of patients with HHT [9]. The diagnosis of PAVM should be suspected in patients with any of the following presentation: (1) one or more pulmonary nodules associated with typical radiographic findings, (2) mucocutaneous telangiectasis, and (3) hypoxemia, polycythemia, clubbing, cyanosis, cerebral embolism, or brain abscess.

Chest X-ray may show one or more rounded multilobular opacities when the fistulas are large enough. Cardiomegaly may rarely be present when the fistulas are very large and cause a hyperdynamic state. However, the chest film is not a very sensitive diagnostic modality. Pulse oxymetry is usually abnormal, with various degrees of desaturation but it may not detect small fistulas. The most sensitive non-invasive screening test for PAVMs, either in context of complex congenital heart disease or in patients with HHT, is contrast echocardiography [10]. Radionuclide testing using Tc99m labeled albumin microspheres is also a useful test, which can provide a quantitative measurement of the right to left shunt by measuring the fraction of the injected dose of microspheres reaching the kidneys [11]. Chest computed tomography may demonstrate PAVMs quite accurately. Recently, contrast enhanced magnetic resonance angiography was compared to helical CT. It was found quite sensitive (75%) and very specific (100%). All significant PAVMs with a feeding vessel diameter>3mm were detected. Thus, contrast enhanced magnetic resonance angiography seems to be a very useful nonionizing and noninvasive procedure for the diagnosis and exact anatomic localization of PAVMs [12]. Despite advances in the techniques mentioned thus far, contrast pulmonary angiography remains the gold standard in diagnosing PAVM, and is usually necessary if resectional or obliterative therapy is being considered [5]. Pulmonary artery pressures are either normal or low because of the decreased pulmonary vascular resistance, especially when the fistulas are large or diffuse.

Three forms of management are available for the various types of PAVMs: (1) transcatheter embolization therapy (using different devices such as coils, detachable balloons, and various devices designed for occlusion of atrial septal defects and patent arterial ducts), (2) surgical approach, including lobectomy, segmentectomy or fistulectomy, redirection of hepatic venous flow to the pulmonary circulation, and heart or heart/lung transplantation and (3) medical therapy (including pharmacologic management) in a few cases.

## Conclusion

The diagnosis of PAVM should be considered in infants with severe cyanosis without structural cardiac lesions or pulmonary hypertension, after excluding other causes of cyanosis, such as parenchymal lung disease and the rare methemoglobinemia, as described before. A high index of suspicion is required for a successful echocardiographic diagnosis of PAVM in a cyanotic infant with the use of contrast echocardiography. Pulmonary angiography is mandatory whenever a diagnosis of PAVM is suspected in order to confirm the diagnosis and a precise identification of the number and location of all lesions before embolization or surgical intervention is undertaken.
